# Enhancing Crop Resilience to Drought Stress through CRISPR-Cas9 Genome Editing

**DOI:** 10.3390/plants12122306

**Published:** 2023-06-14

**Authors:** Gyanendra Kumar Rai, Danish Mushtaq Khanday, Pradeep Kumar, Isha Magotra, Sadiya M. Choudhary, Rafia Kosser, Raviraj Kalunke, Maria Giordano, Giandomenico Corrado, Youssef Rouphael, Sudhakar Pandey

**Affiliations:** 1 School of Biotechnology, Sher-e-Kashmir University of Agricultural Sciences and Technology of Jammu, Jammu 180009, India; ishamagotra316@gmail.com (I.M.); sadiyamaryum785@gmail.com (S.M.C.); roohchouhan123@gmail.com (R.K.); 2 Division of Plant Breeding and Genetics, Sher-e-Kashmir University of Agricultural Sciences and Technology of Jammu, Jammu 180009, India; khandayd2@gmail.com; 3 Division of Integrated Farming System, ICAR-Central Arid Zone Research Institute, Jodhpur 342003, India; pradeep.kumar4@icar.gov.in; 4 Donald Danforth Plant Science Center, St. Louis, MO 63132, USA; rkalunke@danforthcenter.org; 5 Dipartimento di Agricoltura, Alimentazione e Ambiente (Di3A), University of Catania, Via Valdisavoia 5, 95123 Catania, Italy; 6 Department of Agricultural Sciences, University of Naples Federico II, Via Università 100, 80055 Portici, Italy; giandomenico.corrado@unina.it (G.C.); youssef.rouphael@unina.it (Y.R.); 7 Indian Council of Agricultural Research, Krishi Anusandhan Bhavan II, New Delhi 110012, India; sudhakariivr@gmail.com

**Keywords:** abiotic stress, drought tolerance, agriculture, yield, osmotic stress

## Abstract

With increasing frequency and severity of droughts in various parts of the world, agricultural productivity may suffer major setbacks. Among all the abiotic factors, drought is likely to have one of the most detrimental effects on soil organisms and plants. Drought is a major problem for crops because it limits the availability of water, and consequently nutrients which are crucial for plant growth and survival. This results in reduced crop yields, stunted growth, and even plant death, according to the severity and duration of the drought, the plant’s developmental stage, and the plant’s genetic background. The ability to withstand drought is a highly complex characteristic that is controlled by multiple genes, making it one of the most challenging attributes to study, classify, and improve. Clustered Regularly Interspaced Short Palindromic Repeat (CRISPR) technology has opened a new frontier in crop enhancement, revolutionizing plant molecular breeding. The current review provides a general understanding of principles as well as optimization of CRISPR system, and presents applications on genetic enhancement of crops, specifically in terms of drought resistance and yield. Moreover, we discuss how innovative genome editing techniques can aid in the identification and modification of genes conferring drought tolerance.

## 1. Introduction

Changes in climate factors such as precipitation and temperature have a significant effect on the productivity of agricultural crops [1,2]. These factors influence crop growth and health, as well as the yield and quality of crops, in a variety of ways [3]. Besides the well-known damage of droughts and heat waves, higher temperatures can speed up the maturity of crops and, more generally, affect the timing of plant development stages (e.g., flowering and fruit set). This leads to additional ecological problems related to pollinators, nutrient cycling, water availability, and predator-pest interactions. Moreover, climate change is also expected to boost the frequency of environmental stresses because of unpredictable weather patterns and extreme weather events [4]. In addition, since groundwater (a crucial resource for irrigation during droughts) is gradually depleting, it is essential to conduct meaningful research to comprehend how severe weather conditions impact the growth and yield of both irrigated and rain-fed crops [5,6]. Regrettably, although the consequences of global climate change on agriculture have already been confirmed on a global scale, research on innovative methods to develop crops and cropping systems that can mitigate the negative impact of drought is lagging

Recent advancements in genetic manipulation technology have allowed for precise and targeted changes in plant genomes, resulting in the development of next-generation crop breeding strategies [7]. One such tool is the CRISPR (clustered regularly interspaced short palindromic repeats)-Cas system, which utilizes protein-coding genes such as Cas1, Cas2, and Cas9 to modify genomes. CRISPR-Cas9 is considered a more efficient and cost-effective alternative to traditional plant breeding methods, marker-assisted selection, and other genetic manipulation tools, making it an attractive option for enhancing crop resilience in drought-prone environments. Derived from a natural gene-editing mechanism in bacteria, CRISPR/Cas9 technology may minimize issues with social acceptability associated with previous genetic modification approaches that employed transgenes. This approach is expected to facilitate the adoption of genetically modified crops in many countries. Compared to older genome manipulation techniques, such as ZFNs and TALENs, CRISPR/Cas9 is simpler and more efficient, with high precision and low operating costs. As a result, it has become a promising approach for improving the genomes of various plant species, including major crops in agriculture [8]. Multiple plant species, from model plants such as *Nicotiana benthamiana*, *N. tabacum*, and *Arabidopsis thaliana* to certain crop plants, have already benefited from the CRISPR/Cas9 system. Additionally, CRISPR-Cas9-driven multiplexing has been successfully used to target multiple genes in a single organism, making it possible to simultaneously address several stress-sensitive genes in high-yield cultivars that are susceptible to various stresses. This technology has enormous potential for generating crop plants that can withstand multiple stresses thus CRISPR/Cas9 technology is at present favored over all other conventional genome modification technologies due to its higher efficiency and simple implementation. Starting from the fact that the CRISPR/Cas approach has been used effectively to increase resistance to a range of abiotic stresses, including drought, salinity, heat, and nutritional deficiencies in significant agricultural crops, this review aims to summarize the potential applications of CRISPR/Cas9-assisted genome editing strategies in crop plants to mitigate the negative effects of drought on crop growth and yield potential, as well as to explore future uses of this tool for developing crop varieties that can tolerate various abiotic stresses.

## 2. Detrimental Consequences of Drought Conditions on Plant

**Figure 1:**
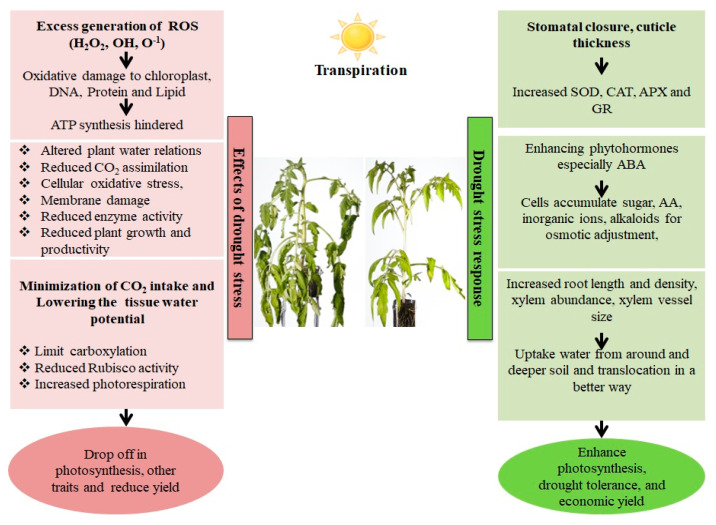
Effect and response of a plant in a drought stress environment. Drought stress causes an imbalance between electron excitation and utilization during photosynthesis, resulting in the generation of reactive oxygen species (ROS), predominantly superoxide (O_2_^−^) and hydrogen peroxide. (H_2_O_2_). These ROS cause oxidative stress by damaging cell membranes, proteins, and nucleic acids. Plants have both enzymatic as well as non-enzymatic detoxification mechanisms to scavenge ROS viz., SOD (Superoxide dismutase), which catalyzes the conversion of O_2_^−^ into the least reactive H_2_O_2_. The H_2_O_2_ is detoxified into O_2_ and H_2_O via the enzymatic activities of Catalase (CAT) and Ascorbate Peroxidase (APX). Non-enzymatic antioxidants involved in cellular defense include carotenoids and glutathione (GSH). Carotenoids defend the photosynthetic machinery byphotoprotection, ROS scavenging, membrane stabilization and contributing to the regeneration of other antioxidants, while GSH protects the chloroplasts from ROS damage bydetoxification, and protection against lipid peroxidation.

Abiotic stresses generated by various climatic conditions can have a detrimental impact on crop development and production. Plants adapt to numerous abiotic challenges by undergoing morphological, physio-biochemical, as well as cellular changes [9]. Drought is one of the major constraints for crop output worldwide because it negatively affects crop efficiency as well as compromising its productivity [10]. Water shortage triggers a variety of crop reactions at physio-biochemical, molecular, as well as morphological planes, inevitably distressing crop yield [11] by impacting different functions, as illustrated in Figure 1. Even a short dry spell negatively impacts plant water dynamics during plant growth, which then disrupts the whole metabolic activity, both at the molecular and physiological levels, depending on the degree and extent of drought [12,13]. When there is a water shortage, one of the primary drivers of the response of plants at the cellular level is indirect or direct oxidative stress. This results in the modification of metabolic machinery as well as the destabilization of membrane stability, which causes extreme metabolic concerns and drastically alters plant activity [14,15]. Drought is acknowledged as a constraint in many aspects of crop production. One of the most basic factors of crop development is germination, which influences overall plant health, and is highly vulnerable to drought. Substantial variations in germination rates have indeed been confirmed for numerous crops, viz., wheat, sorghum, and maize. Further, under the influence of water stress, plant growth is often reduced with or without any sign of leaf wilting in the initial phases of vegetative growth, and flowering can be disrupted [16]. Due to low soil moisture during drought, crops frequently have trouble in absorbing nutrients, which inhibits stem growth; reduction in shoot length was evident under water shortages in the studies [17]. Drought is a limiting factor that impacts several physiological processes in plants, especially metabolism, and proliferation. On the other hand, plant drought tolerance responses are activated simultaneously, encompassing a variety of biological mechanisms that are active at different stages of plant growth and operate somewhere at the level of cells, tissues, and, ultimately, the entire plant. Specifically, plants increase water transport and absorption by growing a much more productive, deep, and wide root system while they prevent water loss by maintaining an optimum rate of transpiration [13]. Additionally, the growth of drought-resilient crops is aided by the employment of crop growth regulators, membrane coherence retention, ideal plant cultivars, antioxidants, proteins related to drought stress, and ion channel proteins such as aquaporins [18].

## 3. Molecular Aspects Underlying Resilience of Crops against Drought

Enhancing agronomic attributes of crops that would provide resistance to multiple abiotic and biotic stresses has always indeed been a worldwide concern [19]. The understanding of global warming and climate change emphasizes the importance of incorporating specific, realistic, and sustainable strategies. Crop yield sustainability amid drought seems to be an important concern in some countries. Drought intensity varies over time and space, so to endure stress; plants have adapted complicated mechanisms with diverse physiological and morphological approaches [20]. Crops confront drought through different degrees of adaptation, avoidance, and evasion [21]. Exploiting genetic characteristics which boost drought tolerance while maintaining high yield is critical during plant management. Drought resistance of wheat, soybean, rice, and maize might be improved using recombinant and classic breeding approaches. Previously, traditional breeding remained the most fruitful method of growing plants, promoting their growth in water-stressed ecosystems. Those very same strategies, however, seem to be labor and time intensive, as well as costly. Under environmental stresses, molecular markers have already played a vital role in attempting to portray plant genetic variability [22]. Several quantitative trait loci (QTLs) associated with boosting drought resilience have been already identified in various crops. However, the precision and reliability of QTL recognition continue to be an issue [23]. Considering this, genome editing seems to have been extremely successful in enhancing crop resistance to abiotic and biotic stresses [24]. Technological advances that can broaden the response of crops to stress as well as make them more environmentally friendly are needed. The advent of genome editing techniques has already resulted in major breakthroughs in plant breeding. Genetic manipulation tools employ sequence-specific nucleases to incorporate recognized variations into the genome [25]. CRISPR-Cas gene-editing systems have attracted widespread praise for their versatility and easiness of use. The whole strategic approach utilizes a guide RNA and an intricate endonuclease (Cas endonuclease) that alters DNA strands to generate double-stranded DNA breaks. Such breaks are then restored via endogenic cellular repair mechanisms, resulting in the generation of novel genetic variations [26]. Competently, the CRISPR-Cas platform has been employed to achieve tolerance against a multitude of abiotic stresses, which include toxicity of heavy metal ions, salinity, drought, and submergence [27]. The ongoing review provides an overview of the use of the CRISPR/Cas9 platform in plants to accomplish drought resistance, as well as explores the technology’s potential towards the increment of drought-tolerant plant varieties. Comprehensive molecular research findings have elucidated to decipher the cellular mechanisms that control plant drought response. Abscisic acid (ABA) modulates plant drought response by limiting stomatal conductance and gene expression to restrict water loss via transpiration [12]. The transcription factor basic leucine zipper (bZIP), also known as ABA-responsive element (ABRE)-binding proteins, is required for ABA signaling [28]. *AREB1* increased expression (*ABF2*) enhanced drought resistance in soybean, rice, and Arabidopsis, whereas AREB1 failure promoted drought vulnerability [29]. Moreover, during drought, *AREB1* regulates a broad array of genes downstream of the ABA signal transduction pathway, including ABA-mediated antioxidant signaling, ABA biogenesis, and osmotic stress response. As a result, *AREB1* is indeed an interesting candidate for improving the responses of plants to drought [30]. The availability of genome sequences for many plants, as well as breakthroughs in gene editing techniques, had already opened new opportunities for breeding for a variety of attractive characteristics. Technological advancements in gene editing, such as transcription activator-like effector nucleases (TALENs) and zinc-finger nucleases (ZFNs), have also enabled molecular biologists to target any desired gene even further precisely [31]. Nonetheless, the above strategies are costly as well as time-consuming since because they involve complex stages encompassing protein designing. Unlike first-generation gene-editing initiatives, CRISPR/Cas9 method includes simple cloning methods and convenient implementation. This very same Cas9 is being experimented with several different guide RNAs to target multiple sites in the genomic DNA. Regarding concrete evidence experiments in plants with a preparatory CRISPR/Cas9 unit, a variety of Cas9 endonucleases (StCas9, SaCas9, and NmCas9) have already been introduced to optimize target precision and decrease off-target cleavage. Moreover, the accessibility of Cas9 enzymes from some of the other bacteria has improved gene editing effectiveness and accuracy [32]. The current review summarizes the crop augmentation options that are available to agro-biotechnologists using well-established Cas9-based gene-editing strategies. Cas9 enzymes have been utilized to improve resistance to biotic and abiotic stresses. Incorporating these techniques is anticipated to result in non-genetically altered (non-GMO) crops with the desired phenotype, which might also increase yield under biotic and abiotic stress conditions [21].

## 4. CRISPR/Cas9-Based Precise Genome Editing for Crop Drought Resilience

**Figure 2:**
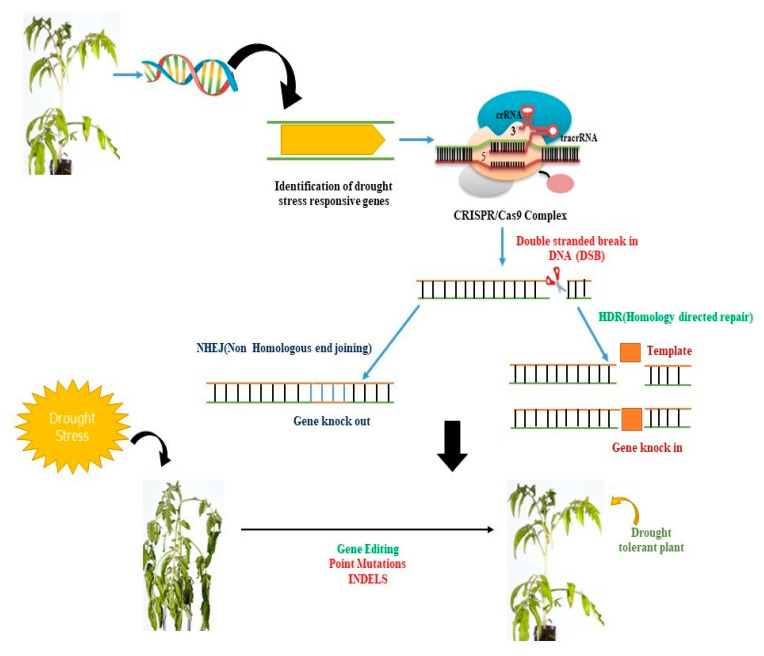
CRISPR/Cas9-mediated genetic manipulation can be used to enhance plant productivity under stress conditions. The Cas9 protein can be guided by a single guide RNA (sgRNA) to a specific genomic region of interest. The CRISPR/Cas9 system then identifies a G-rich protospacer adjacent motif (PAM) region at the proximal end of the target DNA and cleaves it, creating a blunt-ended double-stranded break (DSB). These DSBs can be repaired by the plant’s endogenous repair system via non-homologous end joining (NHEJ) or homology-directed repair. CRISPR/Cas9 can induce mutations through insertions or deletions (INDELs), gene deletions, or multiplex gene knockout, providing a powerful tool for genetic manipulation in plants.

**Figure 3:**
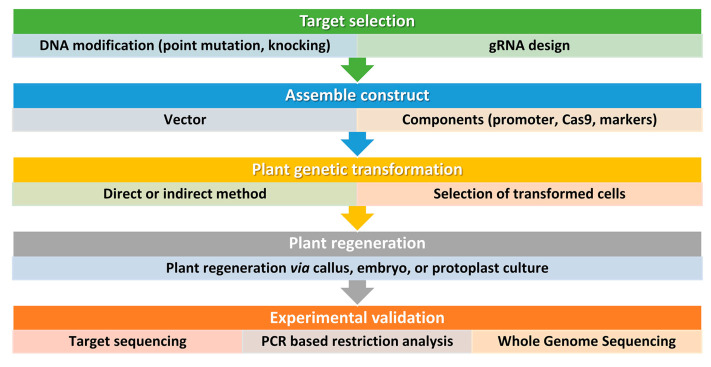
Simplified workflow for CRISPR/Cas9 genome editing in plants. This workflow outlines the key steps involved in using this technology for plant biotechnology. It is important to consider all factors before starting and to design and implement screening procedures also beyond the DNA analysis of the transformed plants. Step 1: Selection of target sequence. The first step in CRISPR/Cas9 genome editing is to select the target sequence. The aim is typically to generate point mutations or small insertions/deletions that result in gene knockout or loss of function. An accurate guide RNA (gRNA) design is carried out to maximize efficiency and minimize the risk of off-target mutations. Step 2: Vector design and assembly. Before constructing a vector, several factors should be considered, including the techniques used for plant genetic transformation and the aim of the study. Several vectors are available and can be tailored for a specific application. For instance, Cas9 and gRNA can be generated from the same vector or separate vectors, and, the Cas9 and gRNA expression can be driven by different promoters according to the plant species and aims. The most used proteins are based on the type IIA Cas9 from *Streptococcus pyogenes*. The native Cas9 coding sequence has been codon optimized for monocots or dicots. Step 3: DNA delivery. Delivering DNA into plant cells is performed using conventional methods in plant biotechnology, such as Agrobacterium-mediated transformation, biolistic microparticle bombardment, or protoplast transformation, followed by plant regeneration when necessary (Step 4). Step 5: Screening. The screening of plant DNA follows standard procedures and may include whole genome sequencing to check for off-target mutations, especially if back-crossing is not a viable option.

Multiple molecular investigations have shown that the ABA is the primary component of plant drought responses, controlling both the expression of target genes relating to stress and stomatal conductance, which prevents water loss. The essential elements of ABA signaling are indeed the binding domain (ABRE) and bZIP unit (AREBs/ABFs) of transcriptional regulators, also known as ABA-responsive factors [33]. Whereas the *AREB1* inactivation increased susceptibility to drought, *AREB1* overexpression increased their ability to withstand it. *AREB1* is a crucial component of the osmotic stress response, antioxidant signaling, and ABA biogenesis [34]. It controls a diverse range of gene expression across the ABA signal transduction pathway. As a direct consequence, *AREB1* might be regarded as a crucial target for boosting plant drought resilience (Figure 2 and Figure 3). To unlock the promoter region of *AREB1* in *Arabidopsis*, a customized CRISPR-Cas9 system combining sgRNA, its catalytic subunit of the HAT enzyme, and dead Cas9 (dCas9) was adopted [35]. Apparently, acetylation of the core histone that resulted from the engagement of the Arabidopsis HAT catalytic site increased the *AREB1* promoter area’s responsiveness to the transcriptional zone. Mutants had greater levels of *AREB1* transcription, stomatal conductance, as well as chlorophyll despite drought, according to physiological and molecular studies. Furthermore, in the presence of water stress, *AREB1* induced *RD29A* transcription [35]. Under drought stress, the recombinant CRISPR lines showed enhanced survival rates.

Overall, these findings show that drought-responsive genes may be positively controlled by the CRISPR-Cas system to effectively elicit epigenetic modifications for improving plant drought tolerance. The primary activator of the ABA-dependent high osmotic stress response and signaling is *SNF1* associated protein kinase 2 (SnRK2), a class of protein kinases peculiar to plants [36]. Seed germination, response to hyperosmotic stress, ABA-mediated stomatal closure, ABA signaling, drought resistance, and plant growth are all processes that are affected by the frequent involvement of SnRK2 members. The activation of drought-regulated genes by *AtSnRK2.8* in *Arabidopsis* really demonstrated a characteristic stress regulatory network to positively influence drought resistance [37]. *AREB-ABF* axis and its receptors were controlled by SnRK2 irrespective of the fact the absence of appreciable changes in stomatal damage as well as mortality seen between the wild-type and the *SnRK*2.8 mutant, according to microarray analysis. Similarly, plant growth improvements and abiotic stress response were shown in rice when *SnRK2* family members of sub-class I and III were present [38].

## 5. CRISPR and Crop Productivity in Drought Resilient Crops

Drought affected plants exhibit reduced plant height, withering leaves, and disturbance in blooms and buds during the plant developmental phases [15]. Drought typically prevents plants from absorbing nutrients. CRISPR/Cas9 was employed in tomato to decrease mitogen-activated tomato protein kinase 3 (*slmapk3*) to elucidate the transcriptional cascade underpinning *slmapk3*-mediated drought resistance (Table 1). Mutants (*slmapk3*) exhibited significantly extensive stem curling and leaf withering during droughts than the control (‘Ailsa Craig’) [15]. Furthermore, the mutant plants had much greater H_2_O_2_, proline, and MDA levels than that of wild type plants, indicating that mutant strains were subjected to much more extreme oxidative stress as well as membrane damage during drought. Subsequent study incorporating gene editing to boost *SlMAPK3* transcription in tomatoes may increase yield as well as drought resistance [15]. CRISPR-Cas9 technology was employed to develop an osmotic stress/ABA-activated protein kinase 2 (*SAPK2*) impairment mutants in rice, which is a critical modulator of ABA signaling. Rice mutant *SAPK2* was considerably more sensitive to oxidative and drought conditions than wild type implying that *SAPK2* is required for drought tolerance in rice and thus could be a promising gene of interest for further crop improvement [39]. Comparable to this, in maize, *ARGOS8-v2* and *ARGOS8-v1* had transcriptional levels that were dramatically higher compared to the wild type, and the ARGOS8 variation had significantly enhanced grain output during drought conditions with minimal yield reduction during normal growing conditions [40]. Scientists subsequently confirmed that *ARGOS8* variations created by CRISPR/Cas9 produced more grain in the fields even during dry season. These results suggest that unique allelic changes may be effectively and efficiently induced using CRISPR-Cas9 technique to create drought tolerant cultivars. A highly effective CRISPR/Cas9 combination employing gRNAs and Cas9 controlled by the tissue-specific promoter *AtEF1* reliably allowed for the effective induction of mutations in genes responsive to abiotic stress (*OST2/AHA1*) without any unintended side effects [41]. The new *OST2/AHA1* mutated alleles in *Arabidopsis* were produced by simplifying CRISPR/Cas9 system with strong stomatal reactions. The use of CRISPR/Cas9 driven genetic manipulation to increase agricultural output as well as multiple genetic stress resistance was made possible by these results [42]

## 6. Implications of CRISPR-Cas9 Promoting Drought Stress Tolerance by Modulating Ethylene Responsive Factors (ERFs)

Ethylene has a significant function in the response towards heat and drought among several phytohormones engaged in several physiological mechanisms driving abiotic stress response [55]. Ethylene participates in signal transduction pathways and is also essential for cell proliferation, germination, senescence, fruit ripening, and stress response [56]. Ethylene does, in fact, play a crucial role in controlling several plant development pathways by mitigating extensive damage. ERFs are activated by salinity or drought stress amid ABA suppression. Several plants, including tomato [57], tobacco, as well as *Arabidopsis thaliana*, have been associated to stress tolerance through the amplification of ERFs-like transcription factors [37]. Genes involved with abiotic stress that control numerous biochemical and cellular processes still carry a biologic significance and CRISPR/Cas9 genome editing approach can be employed to effectively aim these genes. Merely a few researches so far have shown how genome editing for abiotic stress may be used to create crops that are tolerant to global climate change. ERFs are transcription factors that are engaged in a variety of stress-responsive pathways in plants and play significant roles in signal transduction. ERFs constitute transcription factors which are engaged in a variety of stress-responsive pathways in plants and play significant roles in signaling cascades [58]. Utilizing CRISPR/Cas9 system, new variations of *ARGOS8* which is a negative regulator of ethylene response, were created in maize, and mutant lines developed were more drought resistant than wild type. Under field circumstances during the times of drought, the CRISPR/Cas9 edited lines produced more crop yield. Selective change of stress-responsive transcription factors viz., wheat ethylene responsive factor 3 (*TaERF3*) and wheat dehydration-responsive factor binding protein 2 into wheat protoplast were accomplished using CRISPR/Cas9 technology. *OsERF109* was knocked out in rice via RNA interference, considerably enhancing its drought resistance [59]. Comparable to this, the CRISPR/Cas9 approach targeting *ERF* (Ethylene response factors) family members *OsERF109*, *OsBIERF4*, *OsBIERF3*, and *OsBIERF1* may enhance rice’s ability to withstand abiotic stress. Consequently, methods for genetic manipulation can be utilized to increase resistance to a variety of abiotic challenges. Rice varieties that are more robust under abiotic stress are being created faster due to development of novel gene editor CRISPR/Cpf1 [43,57].

## 7. Examples of CRISPR/Cas9 Modified Crops for Tolerance against Different Abiotic Stresses

Abiotic stress response is a complex quantitative feature controlled by numerous genes, making it challenging to manage [60,61,62]. The application of CRISPR-Cas9 technology for targeted gene editing in plants has advanced significantly in recent years. One of the latest tools, CRISPR-P, is a web-based platform that facilitates the construction of sgRNAs in more than 20 different plant species [63]. Additionally, the development of various vectors and support tools for CRISPR-Cas9-based plant genetic manipulation has made it more accessible for applied research [64]. These advances have established the credibility of CRISPR-Cas9 technology for genetic alteration, transcriptome control, stress-resistant crop creation, and molecular studies of multigenic stress response. The type 2 CRISPR-Cas9 approach has enabled precise site-specific alterations in different plant species, including model crops. For instance, in *Oryza sativa*, CRISPR-Cas9 technology targeting *OsDERF1*, *OsERF922*, and *OsRMC* has shown potential for generating stable lines with improved abiotic stress resistance, particularly drought [65]. Similarly, in *Glycine max*, targeting genes *GmMYB118*, *GmDrbza*, and *GmDrbzb* using CRISPR/Cas9 has been proposed as a solution to develop genome-edited lines with improved drought and salt tolerance [66,67]. In *Triticum aestivum*, genes such as *TaDREB2*, *TaDREB3*, *TaHAG1*, and *TaALs* can serve as critical targets for developing wheat cultivars with improved tolerance towards drought as well as salinity, and herbicides resistance [50,52,68]. Furthermore, CRISPR/Cas9 technology can be employed in Brassica napus for editing genes *BnaA6.RGA* and *BnAls* to boost drought as well as herbicide resistance [69]. In *Zea mays*, genes such as *ZmARGOS8*, *ZmALS1*, *ZmALSZ*, and *ZmTMS5* have been for developing genome-edited lines with improved tolerance against drought as well as herbicides and extreme heat tolerance [43,44,70]. In vegetable crops, CRISPR-Cas9 has been often used in *Solanum lycopersicum*, targeting genes such as *SlARF4*, *SlHyPRP1*, *SlBZR1*, *SlcBF1*, and *SlEPSPS* to develop tomato lines with enhanced tolerance to drought as well as range of other abiotic stresses such salinity, heat, cold, and herbicides [71,72,73,74,75,76]. These studies demonstrate the potential of CRISPR-Cas9 technology in improving plant traits and generating stress-resistant crops.

## 8. Conclusions and Outlook

Crop improvement methods including traditional breeding, mutagenesis as well as molecular breeding and transgenics are time-consuming and expensive. Additionally, they can fail in achieving only the desired changes in crop species. Genetic transformation, on the other hand, can create unpredicted modifications, for instance, by insertional mutagenesis, adding a diversity that is not expected to be present in the plant gene pool. The use of genetically modified (GM) crops has sparked controversy and led to temporary suspensions or even prohibitions on their growth by some governments, at time without clearly defined timelines for when such measures will be lifted. Consequently, the potential of the “gene revolution” has been limited. The development of new genome editing techniques like CRISPR offers promise for addressing the challenges associated with GM crops. Removing selectable markers and the Cas9 gene from the plant genome would result in a plant that is similar to one produced using non-genetic engineering methods like mutagenesis. CRISPR can also facilitate efficient and precise multiplex gene editing to develop genome-edited crops that are tolerant to multiple stresses in a single transformation event. Therefore, genetic manipulation through CRISPR is likely to become the preferred method for producing desirable genetically modified crops to address climate change more efficiently [73,76]. 

CRISPR technology has been considered a powerful tool for the development of drought resilient crops, however due to the lack of single, well-defined variations in genes governing inheritance of traits related to drought tolerance could be major obstacle for development of CRISPR mediated drought resilient crops. Furthermore, there are some attributes related to the CRISPR technology that needs further refinement to ensure effective and precise genome modifications in plants. These include selection and understanding of preference and accessibility of Cas enzymes, identification of functional sgRNAs without off-target consequences in the genome, and improvements of the delivery technique of CRISPR ingredients [77]. Newer CRISPR-based techniques are often developed for bacterial and mammalian genome editing before being tested for plant usage [78]. As a result, more improvements are required to allow the deployment of newly emerging CRISPR technologies in different plant species to counter prevailing drought stress. Prime editing, for example, is a potential tool for altering small DNA sequences, although it is currently difficult to utilize in crops due to poor effectiveness [79]. Therefore, more initiatives are required to enhance the prime editing tool for plant usage. Furthermore, present base editing technologies are incapable of installing all sorts of base replacements at specific genomic areas. Another component of CRISPR applicability that must be improved is tools for specifically targeted insertion, which would allow insertions to either stimulate or repress the transcription of downstream genes [80]. Target selections as well as structural characteristics of the sgRNA sequence are indeed important in genetic engineering investigations [78]. To minimize off-targets or failure owing to a variety of possible reasons, the sgRNA could be built using previous information of the genome sequence.

Genetically altered plants, especially those modified with CRISPR/Cas9 technology, mutate their genomes via base pair deletions, substitutions, or insertions, whereas GMOs involve the incorporation of transgene into the organism, which may or may not be integrated into the genome. Considering this fundamental distinction, gene-edited organisms are frequently subject to the same set of rules and restrictions as GMOs in many nations. This means that the significant impediments towards generating GM crops also apply to CRISPR/Cas9-edited crops, which may divert money and investment away from future research on CRISPR/Cas9. Regulations created for earlier technology cannot reasonably be integrated with present technical advances. On the other hand, to keep up with the transformative potential of innovation, laws and regulations must be updated. Therefore, regulations must be changed as appropriate rather than considering outdated GMO regulations as a blanket that cannot continuously cover new and emerging technologies like CRISPR [81]. Besides regulatory and societal concerns surrounding the use of genetically modified crops, a major limitation, common to other biotechnological approach for gene modification, is the inefficiency of plant regeneration after gene editing. In addition, off-target mutations can occur in the genome of the edited plants, which can lead to unintended genetic modifications and negatively impact plant development and function. Moreover, while it is relatively straightforward to generate knock-out and loss-of-function mutants, it is necessary to note that gene editing may not always lead to the desired phenotype or result in a complete loss of gene function and in some cases, it may have unintended effects on other genes or regulatory networks. Finally, achieving precise control over the level of gene expression in the targeted plants can be challenging, particularly for complex traits like drought tolerance that involve the coordinated expression of multiple genes and regulatory networks.

In conclusion, our review highlights the importance of closing the information gap in drought-related signaling pathways to develop crops that can withstand multiple stress factors. The CRISPR/Cas9 system plays a pivotal role in uncovering the biological functions of genes and enables smooth tuning of crop response pathways against drought, salt, and other abiotic stress. In addition, the practical impact of CRISPR/Cas9 in enhancing plant resistance to drought is enormous. With the ability to precisely edit the genome of crops, re-searchers can target specific genes involved in drought response pathways, resulting in plants that are more resilient to drought stress. This technology has the potential to significantly increase crop yields in regions that are affected by drought, ensuring food security for millions of people worldwide.

## Figures and Tables

**Table 1:** Examples of CRISPR/Cas9 driven genetic modification in major crops to alter resilience to drought.

Crop	Gene of Interest	Abbreviation Key	Gene Function	Trait	Reference
Maize	*ARGOS8*	-	Transcription Factor	Enhanced grain yield in filed (under stress conditions)	[43]
Tomato	*SlNPR1*	Nonexpresser of Pathogenesis- Related Genes 1	Transcriptional coactivator	Reduced drought tolerance in the mutants	[44]
	*SlLBD40*	Lateral Organ Boundaries Domain	Transcription Factor	Drought tolerance in the knockout lines	[45]
	*SlMAPK3*	Mitogen-Activated Protein Kinases	Signal transduction	Reduced drought tolerance in the mutants in greenhouse conditions	[46]
Rice	*SAPK2*	Stress-Activated Protein Kinase 2	Signal transduction	Mutants more sensitive to drought stress	[39]
	*OsSRL1*, *OsSRL2*	Semi-Rolled Leaf	Transcription Factor	Drought tolerance (higher grain filling under stress)	[47]
	*OsPYL9*	Pyrabactin Resistance-Like	Transcription Factor	Higher yield under normal and drought conditions (in growth chamber)	[48]
	*OsDSL*	Drought and Salt Tolerance	Transcription factor	High tolerance to NaCl moderate tolerance to osmotic stress at seedling stage	[49]
	*OsRR22*	Response Regulator	Transcriptional regulator/Signaling	Salinity tolerance at seedling stage	[50]
	*OsERA1*	Enhanced Response to ABA	Transcriptional regulator/Signaling	Enhanced response to drought stress	[51]
Wheat	*TaDREB2*	Dehydration Responsive Element Binding protein 2	Transcription factor	Enhanced drought tolerance	[52]
	*TaERF3*	Ethylene Responsive Factor 3	Transcription factor	Enhanced drought tolerance	[52]
Sugar- cane	*ScNLTP*	Non-specific Lipid Transfer protein	Structural gene	Alteration of MeJA-induced pathways	[53]
Soybean	*GmHdz4*	Homeodomain- Leucine Zipper	Transcription Factor	Higher drought tolerance	[54]

## Data Availability

No new data were created or analyzed in this study. Data sharing is not applicable to this article.
